# Agency, Ownership and the Potential Space

**DOI:** 10.3390/brainsci11040460

**Published:** 2021-04-05

**Authors:** Shahar Arzy

**Affiliations:** 1 Neuropsychiatry Lab, Department of Medical Neurobiology, Faculty of Medicine, Hebrew University of Jerusalem, Jerusalem 91120, Israel; Shahar.arzy@ekmd.huji.ac.il; 2 Department of Neurology, Hadassah Hebrew University Medical School, Jerusalem 91120, Israel

**Keywords:** self, experience, mental map, mental model, transition object, Winnicott

## Abstract

The potential space, the space between the experiencer and the experience, is at the heart of Winnicott’s theory. The concepts of agency of one’s actions and ownership of one’s experience have been recently applied to such a space lying in between the experiencing self and the mental (cognitive) map she creates, representing her surroundings. Agency is defined as “the sense that I am the one who is generating the experience represented on a mental map”, while ownership is defined as “the sense that I am the one who is undergoing an experience, represented on a mental map”. Here these concepts are introduced and applied to five main realizations of Winnicott’s potential space: Playing, transitional phenomena, the therapeutic space, culture and creativity. Through theoretical constructs and clinical analyses, it is shown how agency and ownership, and their mutual interrelations, may help to better understand Winnicott’s theory with implications to clinical practice.

## 1. Introduction

“I have tried to draw attention to the importance … of a third area, that of play, which expands into creative living and into the whole cultural life of man. … I have located this important area of experience in the potential space between the individual and the environment … [a space] which both joins and separates”.

(Donald Winnicott, [1], pp. 102–103)

“Perhaps the most important and at the same time most elusive of the ideas introduced by Donald Winnicott is the concept of potential space” (see Appendix), writes the psychoanalyst and interpreter of Winnicott, Thomas Ogden [2]. The potential space may be defined as the intermediate area of experiencing that lies between the inner world and external reality. Central to Winnicott’s life-work of understanding human nature, the concept of potential space is not well understood. In the time elapsed, the scientific concepts of mental models [3,4,5,6] and cognitive maps [7,8,9,10,11] have proven efficient in the representation of the external world. In recent years, the neurocognitive science also progressed much in the study of the human self, its conceptualization and representation [12,13,14,15,16]. However, while these maps, models and concepts enable to represent the perceived world, on the one hand, and aspects of the experiencing self on the other, it seems that the potential space lying in between these two is far less understood. To traverse the potential space, other cognitive operations are required. Here I suggest the senses of self-agency and self-ownership [17], mediating between the self and the world, to account for this traverse. Agency is the sense that “I am the one who is causing or generating an action” [17,18], that is the sense, or operator, that translates the self-originated desire to perform, for instance, a movement to an action. Similarly, agency may represent the sense that “I am the one who is causing or generating an experience” [17,18]. Ownership is “the sense that I am the one who is undergoing an experience”, that is relating the experienced scene (people in the scene, places, events, emotions) to the experiencer [17,18]. In this paper, Winnicott’s potential space as well as the concepts of mental models, agency and ownership are presented. Next, the utility of the agency-ownership operators in better understanding five main embodiments of the potential space is shown [2], including playing, transitional phenomena, the analytic space, culture and creativity.

## 2. The Potential Space

The origin of the term “potential space” may be found in Winnicott’s theory of mother–baby relationships [19,20,21]. According to Winnicott, “Potential space … is the hypothetical area that exists (but cannot exist) between the baby and the object (mother or part of mother) during the phase of the repudiation of the object as not-me, that is, at the end of being merged in with the object” ([1], p. 107). Winnicott suggests that there is a “mental space” between the infant and the mother, the mother represents reality, or the world, while the infant expresses the mental desire or imagination. This space is relevant not only for the infant at that specific stage, but also for the child and even the adult which continues to develop her own potential space in between her own “self” and the world. Moreover, as much as this potential space exists for the infant, it exists for the mother. Later on, Winnicott himself, (as conjugated by Ogden, ([2], p. 129), stated that potential space is an intermediate area of experiencing that lies between (a) the inner world, “inner psychic reality” and (b) “actual or external reality” ([1], pp. 41, 106, 107), that is, the experiencing self and her perception of the environment.

## 3. Agency and Ownership of the Experience

In 2000, the philosopher Shaun Gallagher distinguished between two important concepts: Self-agency and self-ownership (or briefly, agency and ownership; [17,22]). Agency is defined as “the sense that I am the one who is causing or generating an action. For example, the sense that I am the one who is causing something to move, or that I am the one who is generating a certain thought in my stream of consciousness”, while ownership is “the sense that I am the one who is undergoing an experience. For example, the sense that my body is moving” [17]. Following, it has been suggested that in the same manner that agency and ownership help relating movements we initiate and sensations we perceive to our bodily self, agency and ownership are crucial to relate our movement in the environment, which is continuously translated to a form of a map-like representation of the world (“mental-map”), to the narrative (experiencing) self [18]. Notably, agency and ownership thus serve not just as metaphors or a conceptual framework but also as cognitive operators involved in the projection of information to and from the cognitive maps [17,23,24]. Gallagher’s original definitions [17] will therefore be applied such that agency is defined as “the sense that I am the one who is generating the experience represented on a mental-map in my stream of consciousness”, while ownership is “the sense that I am the one who is undergoing an experience, represented on a mental-map”. Agency and ownership therefore carry important information in relating the subjectivity of the experience to the world and back [2,12,25]. To grasp a more coherent picture of agency and ownership of the experience, we shall better understand the other side of the gap, that is the representation of the environment.

## 4. Mental Models and Cognitive Maps

Already in 1943, the psychologist and philosopher Kenneth Craik speculated that “the organism carries a “small-scale model” of external reality and of its possible actions within its head, it is able to try out various alternatives, conclude which is the best of them, react to future situations before they arise, utilize the knowledge of past events in dealing with the present and the future” [9], a speculation that was later extended to the construct of “mental models” [3]. Tolman [10] further hypothesized that perceived stimuli are not connected by “simple one-to-one switches to the outgoing responses”, but rather, are represented on “a tentative, cognitive-like map of the environment. And it is this tentative map, indicating routes and paths and environmental relationships, which finally determines what responses, if any, the animal will finally release” (p.193); decades later, mechanisms have been proposed to support mapping functions mostly in the spatial domain [5,7,8,26]. However, mental models, or maps, are not related only to space. Similarly to spatial maps, temporal maps are crucial for the representation of the relationships between temporally non-contiguous events [27,28,29]. In the 1930s, Bartlett [30] postulated that mental model of the relations in between people, known today as “social network”, is represented similarly to spatial maps. This notion is now further developed both with respect to cognitive mapping as well as network analyses of social maps [31,32]. The potential space is therefore situated between the experiencing self and the representation of the world as “mental-maps”, which in turn may represent places (spatial maps), events (time), people (social network), and maybe also more abstract concepts [33,34,35,36].

## 5. Agency and Ownership on Mental Maps

**Figure 1:**
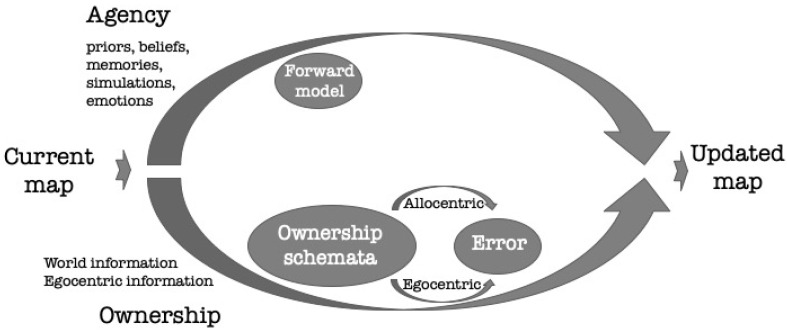
The agency-ownership model (modified from [18]). The agency (**upper**) arm uses self-originated agency-derived priors and beliefs, as well as the current mental map held by the self to predict the next version of the map using a forward model. The ownership (**lower**) arm uses one’s personal model of ownership (schemator) and egocentric–allocentric translation (the difference between the predicted allocentric map and the actual egocentric feedback perceived by egocentric and world-information) to correct the predicted map. The updated map (**right**) is dependent on these two processes.

How are mental maps created and updated by the experiencing self, on the one hand, and influencing this self on the other? The experiencing self carries beliefs, reflections and information that are continuously affecting perception of the surrounding and its further processing [37]. This “subjectivity” (ranging from a simple “I-ness” by which experience is subtly endowed with the quality that one is thinking one’s thoughts and feeling one’s feelings to a more developed state of intentional self-reflection [2,25,38]), influences the mental maps representing the environment. This is represented in the agency-arm of the agency-ownership model (Figure 1, upper arm). Forward-model [39,40] may take the map from a certain state *i* to a new state *i +* 1 as based on the available information (such as beliefs, imaginations, plans or simulations). In addition, the experiencing self also affects perception: Each subject perceives the world differently, developing schemata [41,42] that mediate between the concept one holds regarding the world and its actual perception [43]. These schemata are influenced by the cognitive operator (or “schemator”) that shapes the way in which the experiencer “encounters” the world and integrating new information into the mental map representing the world. Moreover, within the updating process, each subject corrects detected errors between perceptions originated from two different sources—egocentric and allocentric. According to the agency-ownership model (Figure 1) the measured information may be egocentric (encountered by the subject) or allocentric (derived from an external source). Differences between the egocentric and allocentric information sources are subjected to corrections according to the difference between the estimated and the measured information. This is represented in the ownership-arm of the model (Figure 1, lower arm). A similar mechanism also helps in the interaction between the agency and ownership arms.

The most basic realization of the model is in the spatial domain. Expectations regarding the environment (e.g., size, topology, structure, routes [44]) are inserted to the maps through the agency-arm. World information regarding different landmarks, as well as the experience of walking the environment (distance walked in a certain direction, so-called “path-integration”) [45,46] are inserted through the ownership-arm. Performance is dependent on one’s navigation skills, as expressed in the ownership-schemator, and the error correction gain applied [47]. Agency will be defined as “the sense that I am the one who is navigating the environment, represented on the map”, while ownership is “the sense that I am the one who navigated this route, using path-integration, landmarks and a recent cognitive-map”. In the time domain [27], agency may be defined as “the sense that I am the one who is simulating this future or remembering this past scenario”, and therefore inserting memories and simulations to the experience. Ownership is “the sense that I am the one who underwent (or will undergo) this experience, combined by these events”. Agency on the social cognitive map [48] will be defined as “the sense that I am the one who generates and evaluate interactions with people around me (or social network)”. Ownership may be “the sense that I am the one who is involved in a social network” [32]. More generally, the narrative-self (or experiencing-self) refers to the world through life-stories: In this manner, agency may be defined as the sense that “I am the one who tells the story” and ownership as the sense that “I am the protagonist of the story” [18]. 

## 6. Agency and Ownership of Mental Maps and the Potential Space

Agency and ownership are mediating between the experiencing self and the representation of the external world, that is in between the two banks of Winnicott’s potential space. Winnicott mentioned five main phenomena that happen in the potential space: Playing, transitional phenomena, the intersubjective analytical process, creativity and culture ([1] p. 99). In the following, the way in which this mediation is effectuated is detailed for each of these five domains, exemplified by clinical vignettes.

### 6.1. Playing

It is difficult to overestimate the importance of playing in Winnicott’s thinking. “It is in playing and only in playing”, he writes, “that the individual child or adult is able to be creative and to use the whole personality, and it is only in being creative that the individual discovers the self” ([1], p. 54). Playing is happening within the potential space, because, by definition, playing mediates in between the real world and the (playing) self. This includes masquerading, role-playing, pretending, theory of mind (guessing actions of a counter-player), symbolizing and creativity [2,49]. The self does not arrive tabula rasa to the play but carries with her many different “priors” which are part of the player’s personality, characterize her general state (e.g., general feeling during the playing day), or specific emotion evoked by the play. In the same vein, each player has a specific ownership-schemator, which processes information acquired during playing, and a personalized error detection threshold and correction mechanism. To reiterate, to exploit the role of playing in bridging the potential space it is crucial to have a sense of agency, “the sense that I am the one who is playing”, with all the “priors” oneself carries. It is not less important to have the reciprocal feeling of ownership, “the sense that I am the one who is undergoing the playing experience”, a self who has a certain way to process the world and relating the ongoing experience to it. 

A situation brought by Ogden [2] may help to examine the role of agency and ownership as a conceptual explanatory framework, in the context of playing:

A two and a half-year-old child after having been frightened by having his head go underwater while being given a bath, became highly resistant to taking a bath. Some months later, after gentle but persistent coaxing by his mother, he very reluctantly allowed himself to be placed in four inches of bath water. The child’s entire body was tense; his hands were tightly clamped on to his mother’s. He was not crying, but his eyes were pleadingly glued to those of his mother. One knee was locked in extension while the other was flexed in order to hold as much of himself out of the water as he could. His mother began almost immediately to try to interest him in some bath toys. He was not the least bit interested until she told him she would like some tea. At that point the tension that had been apparent in his arms, legs, abdomen, and particularly his face, abruptly gave way to a new physical and psycho–logical state. His knees were now bent a little; his eyes surveyed the toy cups and saucers and spotted an empty shampoo bottle which he chose to use as milk for the tea; the tension in his voice shifted from the tense insistent plea, “My not like bath, my not like bath”, to a narrative of his play: “Tea not too hot, it’s okay now. My blow on it for you. Tea yummy”. The mother had some “tea” and asked for more. After a few minutes, the mother began to reach for the washcloth. This resulted in the child’s ending of the playas abruptly as he had started it with a return of all of the initial signs of anxiety that had preceded the play. After the mother reassured the child that she would hold him so he would not slip, she asked him if he had any more tea. He does, and playing is resumed.

**Figure 2:**
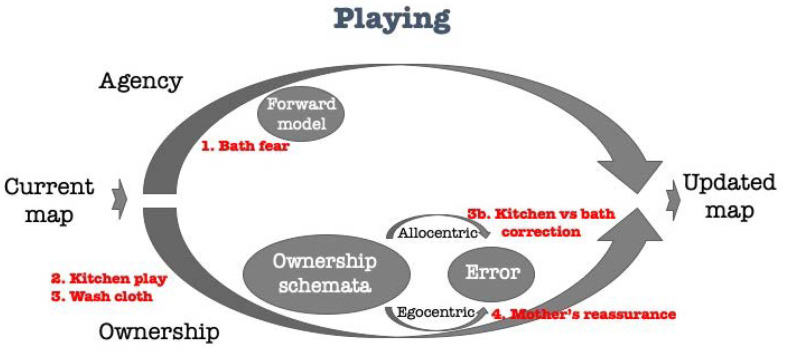
A case of a therapeutic playing [2] explained through the agency-ownership model.

Ogden uses this observation to illustrate how a state of mind was generated by the mother and child, in which the water has been transformed from a frightening experience to a play. This observation may be further understood through the agency-ownership model of the experience (Figure 2). In the initial condition, the bath situation is perceived as a life-threatening event. This is a “prior” concept integrated into the child’s agency operator. The agency-arm receives a relatively high (fear induced) gain and therefore significantly influences the produced mental map. This map is then inserted—in the next cycle—also to the child’s ownership operator (the sense that I am now undergoing this bath experience) through the ownership-arm. The mother then presents a new scenario—this of the “kitchen-play”. This scenario, met and first processed through the ownership-arm, carries for the child a prior of a positive, pleasant value, that is inserted in the next cycle through the agency-arm (though with a lower gain), and the mental map is updated accordingly. He is then introduced to the washcloth (world-information inserted into the ownership-arm). This evokes a bath-related ownership schema and error correction towards the bath scenario with a high gain, enabling an abrupt change in the outcome. The mother then reassures him by empowering the sense of holding, thus inserting more egocentric information into the ownership-arm, and in parallel resumes the positive, slower process of play (asking him for more tea): “He does and playing is resumed”.

### 6.2. Transitional Object and Phenomena

The “transitional object” is probably Winnicott’s most well recognized contribution. Winnicott notes that he “introduced the terms “transitional objects” and “transitional phenomena” for designation of the intermediate area of experience, between the thumb and the teddy bear, between the oral erotism and the true object-relationship” [50]. In between the experiencing-self and the experienced reality lies the experience itself. This third entity is critical since, on the one hand, it distinguishes the self from reality, but on the other hand maintains their mutual relations. Winnicott further extended his observations regarding the transitional object to a more general concept of transitional phenomena, “an intermediate state between a baby’s inability and his growing ability to recognize and accept reality” [50]. This recognition may be related to a sense of agency, the sense that “I am the one whose ability to recognize reality is developed”, complementing a sense of ownership, “I am the one undergoing a transitional phenomenon” (or “holding a transitional object”). 

To illustrate a typical use of transitional object, Winnicott [50] describes the case of Y:

Y has developed in quite a straightforward way throughout. He now has three healthy children of his own. He was fed at the breast for four months and then weaned without difficulty. Y sucked his thumb in the early weeks and this again “made weaning easier for him than for his older brother”. Soon after weaning at five to six months he adopted the end of the blanket where the stitching finished. He was pleased if a little bit of the wool stuck out at the corner and with this he would tickle his nose. This very early became his “Baa”; he invented this word for it himself as soon as he could use organized sounds. From the time when he was about a year old he was able to substitute for the end of the blanket a soft green jersey with a red tie. This was not a “comforter” as in the case of the depressive older brother, but a “soother”. It was a sedative which always worked. This is a typical example of what I am calling a *Transitional Object*. When Y was a little boy it was always certain that if anyone gave him his “Baa” he would immediately suck it and lose anxiety, and in fact he would go to sleep within a few minutes if the time for sleep were at all near. The thumb-sucking continued at the same time, lasting until he was three or four years old, and he remembers thumb-sucking and a hard place on one thumb which resulted from it. He is now interested (as a father) in the thumb-sucking of his children and their use of “Baas”.

**Figure 3:**
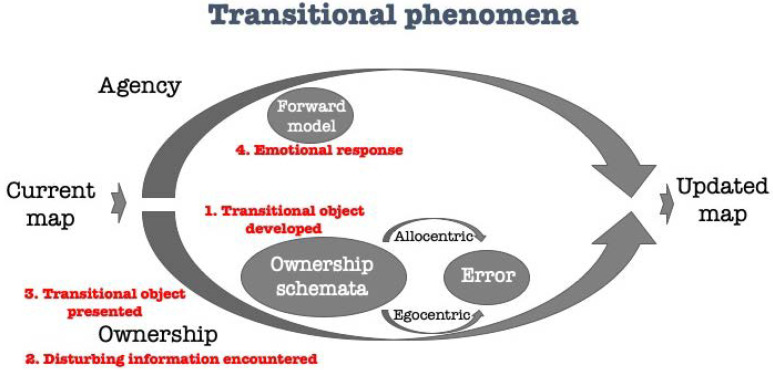
Transitional phenomena and the role of transitional object explained through the agency-ownership model.

As every infant, Y is mediating his way in between the external world and his developing self. Y is clearly helped by transitional objects. The first object is the breast, that according to Winnicott is initially perceived as part of the self yet while weaned is identified as a part of the external world. This, in the agency-ownership model, is embedded in the ownership-schemator (Figure 3). Following is the thumb, which is used for similar goals: The thumb is part of the child’s psyche-soma [51], yet carries some characteristics of an object. Soon after, the blanket is used as mediator, and this time the mediator is also named, facilitating further use. The blanket is then replaced or joined by a jersey, and used for the same goals (soothing, sedation, tranquilizing). Each time when disturbing circumstances are approached (e.g., meeting a stranger, that is a world information through the ownership-arm which evokes specific emotional priors inserted by the agency-arm), the transitional object is presented (to the ownership-arm), evoking positive feelings (through the agency-arm) to overcome the initial negative ones and calm the child.

### 6.3. The Analytic Space

The analytic space is the intersubjective space, in between the analyst and the analysand, two people who try, it may be assumed, to get into a Buberian “I–Thou” relationships [52,53]. I–Thou relationships are mutual relations between two subjects, unlike the subject-object I–It one. To illustrate the difference between the I–Thou and the I–It relationships, Buber [54] tells a story from his childhood experience: “When I was eleven years of age, spending the summer on my grandparents’ estate, I used, as often as I could do it unobserved, to steal into the stable and gently stroke the neck of my darling, a broad dapple-gray horse … what I experienced in touch with the animal was the Other, the immense otherness of the Other … and yet it let me approach, confided itself to me, placed itself elementally in the relation of Thou and Thou with me. The horse, even when I had not begun by pouring oats for him into the manger, very gently raised his massive head, ears flicking, then snorted quietly, as a conspirator gives a signal meant to be recognizable only by his fellow-conspirator; and I was approved.” (p. 11). More concretely, Buber summarizes [53]: “The world of It is set in the context of space and time; The world of Thou is not set in the context of either of these; The particular Thou, after the relational event has its course, is bound to become an It. The particular It, by entering the relational event, may become a Thou” (p. 33).

In an influential paper, Ogden [52] applies a similar approach to the analytic process: “Contemporary psychoanalytic thinking”, he writes, “is approaching a point where one can no longer simply speak of the analyst and the analysand as separate subjects who take one another as objects”. Following Winnicott’s classification of the “experiential space” to self, world (reality) and the experience, Ogden [52] postulates that “The analytic process reflects the interplay of three subjectivities: That of the analyst, of the analysand, and of the analytic third.” This third, he suggests in a Buberian manner, “is a creation of the analyst and analysand, and at the same time the analyst and analysand are created by the analytic third (there is no analyst, no analysand, and no analysis in the absence of the third).” In-line with the agency-ownership model, Ogden mentions that the analytic third is experienced by both the analyst and analysand with respect to priors taken from their own past and personality structure inserted by the agency-arm. Notably, the ownership-schemator similarly perceives “reality” (inputs of world-information, subjective movement (“path-integration”) and pervious maps) in a personal manner, specific to the experiencer, and applies gains and corrections accordingly.

**Figure 4:**
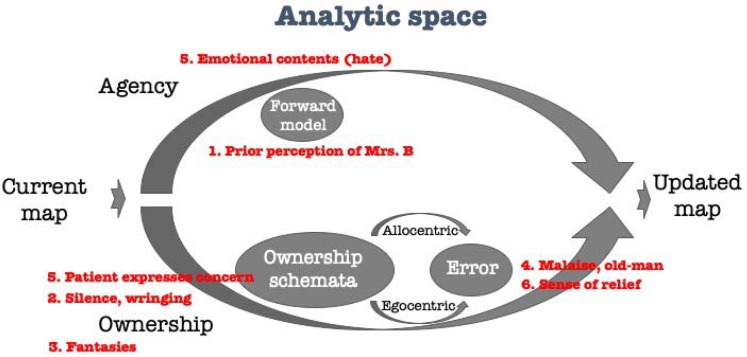
The therapeutic plane: Ogden’s interaction with Mrs. B according to the agency-ownership model.

For illustration, Ogden [52] details the story of an analysis of a patient of him, Mrs. B. Mrs. B was a 42-years-old married lawyer with two children, who started analysis for an ill-defined reason that “she had felt vague discontent with her life”. This first description of the patient already influences the mental model representing her in the analyst’s mind, via the agency-arm (Figure 4), as a patient who suffers from relatively mild difficulties. The analyst then faces several perceptions of the patient (“world-information”) through long periods of silence (~15–20 min each) as well as a continuous hand-wringing. Following, he finds an egocentric (“path-integration”-like in the model) perception that his “own fantasies and day-dreams were unusually sparse during this period of work.” This experience further influences the process such as the mental model of the patient is as the analyst “experienced less of a feeling of closeness to Mrs. B” [37]. This is further translated in the analyst to a flu-like feeling—with malaise, nausea and vertigo, and then to an even more general impression in the analyst of himself as “a very old man”. The bodily feelings may be the result of an inability of the gain function [47] to compensate well for the analyst’s perception (or hate) of the patient [55]. Note that the agency-arm as well may introduce (1) a general feeling which is not necessarily related to the specific patient herself, (2) a general feeling of the patient while encountered (here is negative in general), and (3) concrete impressions of the analyst while at the current situation. 

The analyst then approached an unusual occurrence in the analytic process where “Mrs. B startled me by abruptly (and for the first time in the analysis) turning around on the couch to look at me. She had a look of panic on her face and said, ‘I’m sorry, I didn’t know what was happening to you’.” The agency-arm and the ownership-arm are cross-talking, updating the map again—“I now understood that for several weeks I had been emotionally consumed by the unconscious conviction … that I had a serious illness, perhaps a brain tumor, and that during that period I had been frightened that I was dying.” The map is updated and the analyst “felt an immense sense of relief at this point in the meeting as I came to understand these thoughts, feelings and sensations as reflection of transference–countertransference events occurring in the analysis.” Following this sequence of feelings and emotional changes, Mrs. B herself starts talking. For instance, she details that “she was sorry that she had ever ‘got into this thing’ (the analysis) and wished she could ‘erase it, make it never have happened’.” This leads to another update in the analyst’s “mental map” of the patient: “During these exchanges, for the first time in the analysis, I felt that there were two people in the room talking to one another.”

### 6.4. From Illusory Experience to Cultural Experience, Art and Religion

There are few concepts as influential in human history as Messianism. The concept of a Messiah, a human realm who is destinated to advance the world into redemption, has influenced specific, notable individuals, as well as the life of so many others (through other individuals who underwent a messianic experience). The internal experience of being a Messiah may be perceived differently in different contexts. In the religious context, perceiving a past figure like Jesus as a Messiah is considered as a normal behavior. The experience of *being* one of those figures is, though, a common delusion [56] and a hallmark of schizophrenia. Nevertheless, the cultural, art and religious experience may enable such identifications. For instance, a painter who paints Jesus in a similar manner to an auto portrait will not be considered delusional, as self-elements within a portrait are a hallmark of art. Likewise, in 1500 the famous German painter Albrecht Durer has painted his self-portrait as Christ, as have done afterwards other notable painters like Perugino, Rembrandt, van Dyck or Titian. With respect to religious experience, in the Jewish tradition, for instance, the Messianic mission is not limited to the founder of the religion. Likewise, notable Jewish figures like Rabbi Chaim ibn-Attar in Morocco or Rabbi Abraham Isaac Kook in Palestine have perceived themselves as Messianic figures, as did many other messianic mystics [57]. Moreover, in the teaching of Hassidism, an influential 18th century east-European messianic Jewish movement, every single person who follows the commandments is taking part in the Messianic mission [58,59]. These examples are dependent on the cultural environment in a specific place and time [60]. Likewise, such painters of religious figures may be perceived as sinners and may be executed in one cultural or political scenario, admired in another, or subjected to academic research, but no one will relate to them positive symptoms of schizophrenia.

Winnicott [50], when referring to illusion, which is at the basis of the initiation of experience, states: “This early stage in development is made possible by the mother’s special capacity for making adaptation to the needs of her infant, thus allowing the infant the illusion that what the infant creates really exists” (p. 97). It is this strange phenomenon, namely the mother allowing the infant the illusion that what the infant creates really exists, that distinguishes illusion from material reality. Furthermore, in one of his descriptions of the gap between the mother and the baby Winnicott writes: “I am here staking a claim for an intermediate state between a baby’s inability and his growing ability to recognize and accept reality. I am therefore studying the substance of illusion, that which is allowed to the infant, and which in adult life is inherent in art and religion, and yet becomes the hallmark of madness when an adult puts too powerful a claim on the credulity of others, forcing them to acknowledge a sharing of illusion that is not their own. We can share a respect for illusory experience, and if we wish, we may collect together and form a group on the basis of the similarity of our illusory experiences. This is a natural root of grouping among human beings” ([1], p. 14). Winnicott refers here to three realms of an illusion regarding the gap between the “inner reality and external life”: (1) The illusion held by the infant; (2) illusion held by adults in art and religion; (3) “madness”. The difference between these three may be better understood through its representation on the agency-ownership model.

**Figure 5:**
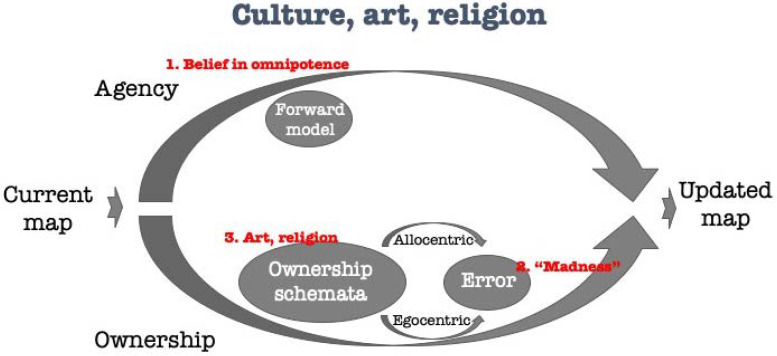
Winnicott’s omnipotence illusion and “madness”, as well as the roles of art and religion in the processing of illusions according to the agency-ownership model.

Illusion takes a central role in Winnicott’s theory [61]. Early on, the infant holds a prior illusionary belief in her omnipotence power regarding the world. This illusion, which is considered as a normal development, dictates the infant’s experience, and is inserted through the agency-arm (Figure 5). On the other hand, there are delusions which are defined as “madness”. Madness is an inherent disturbance within the system. Previous authors suggested that schizophrenia is primarily a disorder of agency, since contents that do not exist in reality (delusions) are inserted into the cognitive system [25,62,63,64]. The problem may be, however, found as well within the ownership-arm, either in the ownership-schemator which may create deviating schemata, or in the gain applied for error corrections. In daily experience, as is also expressed by the agency-ownership model, there is an inherent difference in between perception and estimation. This difference is corrected by the agency and ownership operators with a certain gain applied [47], giving rise to a new updated map. When there is a dysfunction in gain applied, the gap overpasses a certain threshold, and the system’s product is perceived as a delusion (“madness”). It is in the arts or religious experience, as exemplified above, where such products (through designated ownership-schemator) are acceptable. Moreover, beliefs which are apparently accepted by the community (as artistic or religious practice/spirituality) may further enter the process via the agency-arm. That way, these beliefs also allow to enlarge the error between the ownership-schemator and the egocentric experience as these is further corrected while encountered with the agency-arm.

### 6.5. The Area of Creativity

Creativity takes a highest place in Winnicott’s theory: “It is creative apperception, more than anything else, that makes the individual feel that life is worth living” ([1], p.65) he writes. Ogden [2] further locates the creativity process to the potential space: “That space between symbol and symbolized, mediated by an interpreting self, is the space in which creativity becomes possible and is the space in which we are alive as human beings, as opposed to being simply reflexively reactive beings. This is Winnicott’s potential space” (p. 133). Winnicott further identifies creativity as a product of the internal world, unlike compliance with the external world which is uncreative: “Contrasted with this is a relationship to external reality which is one of compliance, the world and its details being recognized but only as something to be fitted in with or demanding adaptation. Compliance carries with it a sense of futility for the individual and is associated with the idea that nothing matters and life is not worth living. In a tantalizing way many individuals have experienced just enough of creative living to recognize that for most of their time they are leaving uncreatively, as if caught up in the creativity of something else, or of a machine. The second way of living in the world is recognized as illness in psychiatric terms. In some way or other, our theory includes a belief that living creatively is a healthy state, and that compliance is a sick basis of life” ([1], p. 65). 

**Figure 6:**
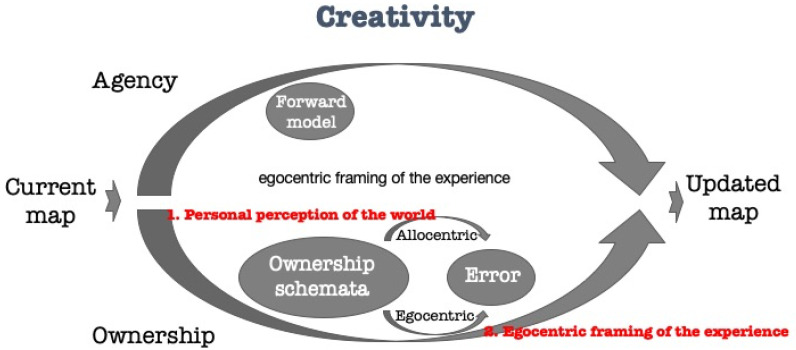
Creativity (and uncreative) processes according to Winnicott through the agency-ownership model.

Winnicott defines the (mentally) “sick” state (a spectrum ranging from health through schizoid state to schizophrenia) of “compliance” as a lack of creativity, or specifically recognition of the world, yet only as something that requires further adaptation. According to the agecy-ownership model (Figure 6) the problem he describes is located in the ownership-arm, responsible to the recognition of the world. More technically and specifically, there may be a disfunction of the ownership-schemator, which is the personalized mechanism perceiving world-information and egocentric perceptions and updating the perception of the world by the individual in a personalized manner.

## 7. Neuroanatomical Considerations

Agency, ownership and mental maps are all based on already established functional neuroanatomy basis (see [5,7,65,66,67]). The processing of the potential space, has recently gained scientific basis with the advances in research of the mother–infant relationships. Studies have shown that not only genetical factors affect the embryo and infant development but also external factors, including socio-emotional ones [68]. Moreover, these may carry a longstanding impact as, for instance, changes in the levels of neurotrophic factors during critical developmental stages might result in long-term changes in neuronal plasticity and lead to increased vulnerability to aging and to psychopathology and to diverse forms of psychopathology in young adulthood [68,69]. In this vein, it has been suggested that the inter-relations created together by the mother and infant, as discussed here, directly affects gene–environment interactions and, moreover, that these relations has thus long-enduring effects [70].

The processing of the human self, which bridges these concepts and constructs, has become central to the functional neuroanatomy research through the discovery of the default mode network (DMN), a network of brain regions involved in self-related activities and internal mentation [71,72,73,74]. Specifically, within the DMN, two anterior regions—the medial prefrontal cortex (mPFC) and the anterior cingulate cortex—are hypothesized to be involved in a self-referential mental activity of the narrative-self [72,73,75,76]. On the other bank of the potential space, mental (or cognitive) maps of the external world are situated in another subsystem of the DMN, the medial temporal lobe (MTL) structures, as based on well designated cell populations [7]. 

**Figure 7:**
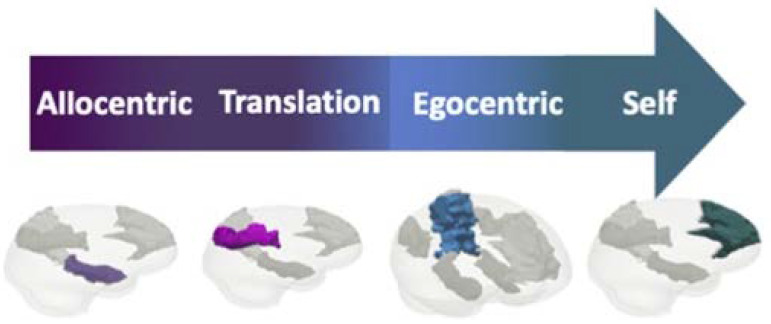
Neuroanatomy of the agency-ownership model. The flow from allocentric representation through agency and ownership to egocentric representation and the self is shown, in parallel to the corresponding brain regions (medial temporal lobe, posterior cingulate/retrosplenial cortex, lateral parietal and temporal cortex, and medial prefrontal cortex, respectively; modified from [18]).

The relations between cortical regions related to agency and ownership, and specifically the different parts of the DMN were matter for a longstanding debate. Experimental studies in the bodily consciousness domain have provided evidence relating the temporoparietal junction (TPJ) at the lateral cortex to agency and ownership processes [77,78,79]. In a meta-analysis [65] Sperduti and colleagues found converging activations including the TPJ, pre-supplementary motor area (SMA), precuneus and mPFC in external-agency, while only the insula was activated during self-agency. In contrast, Renes and colleagues [80] suggested that the experience of self-agency was associated with increased activation in the inferior parietal lobule (IPL, the parietal part of the TPJ) as well as the bilateral (medial) superior frontal cortex and medial prefrontal cortex. Zito and colleagues [81] identified decreased activation during motor control in the TPJ bilaterally only, thus dividing in between areas of “decreased agency” and those of “normal agency”. Pre-SMA and dorsal parietal cortex was found to be causally related to the sense of agency already in the planning phase before movement initiation [82]. To reconcile these different findings and specifically distinctions in a recent series of studies using meta-analysis, Zapparolli, Seghezzi and colleagues [66,67] compared the neural features of body ownership and sense of agency. They identified network specific to the sense of agency at the left SMA, the left posterior insula, the right postcentral gyrus, and the right superior temporal lobe; network for body-ownership at the left IPL and the left extra-striate body area (EBA). The left middle insula was active for both agency and ownership. The mostly distinct networks with minimal overlap have led the authors to propose a model that dissociates between these two functions, yet with a partial co-activation. Taken together, this data suggests a posterior-anterior stream of activities from ownership at the IPL-part of the TPJ as well as the EBA, advancing to agency at the SMA, insula, post-central gyrus and the temporal part of the TPJ, and the self most anteriorly at the mPFC (for allocentric to egocentric processing at the system see Figure 7). Agency and ownership therefore mediate between the two shores of the potential space not only in the cognitive-conceptual plane but also in the mechanistic-neuroanatomical one (see also [73,83,84]).

While the neuroanatomical basis of playing is still to be understood, and furthermore culture, transition phenomena or psychodynamic process, the cognitive neurosciences have recently started to shed light on one of Winnicott’s components of the potential space, that of creativity [83,84,85,86]. Performing a cognitive task requires a combination of both internal thoughts and external perceptions, therefore considered as “dual-tasking” [87], a skill that may be critical for creativity. Creativity may be therefore defined as the ability to cross the potential space back and forth, as based on a well-defined large-scale brain network. Likewise, it was found that functional connectivity in between the self-related DMN and the externally directed executive control (fronto-parietal) network, reliably predicts creative thinking (see [85]). Further advancements in the cognitive neurosciences may help to better understand creativity as well as the other components of the potential space.

## 8. Conclusions

Unlike several other, more speculative, psychological theories, Winnicott’s ideas are well grounded in rich phenomenological and clinical observations, giving his original ideas some justification to further conceptualize psychological phenomena and mental disorders. Analysis of Winnicott’s “potential space” through the neurocognitive operators of agency and ownership in the mental mapping process enabled us to structure, formulate and better understand five main themes in Winnicott’s theory and their implication in clinical practice. This exemplifies how advances in modern cognitive neuroscience may further extend previous theories on the one hand and profit by these theories on the other. Clinicians may use the proposed model to further structure their clinical insights into Winnicott’s model [88,89]. Specifically, the creation of the potential space contributes to the patient’s coming to a new understanding of her/his experience (“updated map”). Patients may therefore become aware for the mutual influence of their own history, memory and beliefs on the one hand through the agency-arm, as well as the potential of planning, imagination and “re-biography”. On the other hand, new encounters, world/allocentric information or personal/egocentric “wandering” may influence this new map through the ownership-arm. Another important component of the suggested model is the weighting (gain application) procedure applied where the agency and ownership arms or the allocentric and egocentric ones encounter each other. Dysregulation of these gain application and/or error detection procedures may lead to pathological processing and may be subjected for analysis. These processes may also be applied to the playing, analytic or cultural processes to facilitate the understanding of the intrapsychic and interpersonal experience. Further research may shed new light on the role of the potential space in human cognition as well as its cognitive, computational, neuronal and clinical implications.

## Appendix

**Glossary**	
Agency	the sense that “I am the one who is generating an experience represented on a cognitive map”. The agency process may insert different priors or beliefs one has with respect to the represented environment.
Cognitive map	a schematic-like mental representation of the relationships between entities in the world including places, events, people, or even concepts.
Mental model	a small-scale representation of the external world and the relationships between its various parts as well as the individual who represents them. A mental model enables an individual to use past experiences to predict future ones.
Ownership	the sense that “I am the one who is undergoing an experience, represented on a cognitive map”. This implies an “ownership-schemator” that processes newly acquired world- and self-related information in an individual manner.
Potential space	an intermediate area of experiencing that lies between imagination and reality. Specific forms of potential space include the play space, the area of the transitional object and phenomena, the analytic space, the area of cultural experience, and the area of creativity.
Psyche-soma	the unification of the body with an imaginative elaboration of somatic parts, feelings, and functions.
